# Editorial: The benefits and challenges of pets for adults with disability or long-term health conditions

**DOI:** 10.3389/fpsyg.2024.1376016

**Published:** 2024-03-06

**Authors:** Jessica Bibbo, Cathleen M. Connell, Polly Yeung, Carol Opdebeeck

**Affiliations:** ^1^Benjamin Rose Institute on Aging, Cleveland, OH, United States; ^2^School of Public Health, University of Michigan, Ann Arbor, MI, United States; ^3^School of Social Work, College of Health, Massey University, Palmerston North, Manawatu-Wanganui, New Zealand; ^4^Department of Psychology, Faculty of Health and Education, Manchester Metropolitan University, Manchester, North West England, United Kingdom

**Keywords:** pets, adulthood (18 years and older), aging, human-animal bond, human-animal interaction (HAI)

Living with a pet is a common and highly sought after form of human-animal interaction. However, the role of pets in the daily lives of adults remains understudied. Much of the empirical literature focuses on animal-assisted interventions or the impact of pets on specific groups (e.g., children, young adults, older adults, residents in long-term care facilities). The overall aim of this Research Topic was to focus on the everyday experiences of pet ownership in adulthood. The four original papers in this issue address how factors at the individual and societal levels shape pet ownership.

McLennan et al. provide an overview of volunteer-based community programs that provide assistance with pet care to older adults and a qualitative analysis of feedback from program participants. Results of the paper highlight how social determinants of health impact people and their pets. Indeed, the lives of people and pets are “intertwined” – both the barriers they face and opportunities for a shared quality of life. The authors provide innovative recommendations for practice, including services and resources that support the bond between pets and people, and discuss ways to foster collaboration across community sectors.

Merkouri et al. conducted a theoretically based mixed-methods study to provide a nuanced picture of dog ownership and wellbeing of adults across the United Kingdom. Validated measures were employed to investigate the associations between specific aspects of the dog-owner relationship and wellbeing outcomes. The thematic analysis uncovered specific ways a relationship with a dog can influence both hedonic and eudaimonic wellbeing. The paper provides important insights on facilitating opportunities and challenges due to the responsibility of pet ownership.

Bibbo et al. surveyed an interdisciplinary sample of professionals who work with older adults and caregivers to examine a previously unexplored aspect of how pets impact daily life. They discovered that the majority reported encountering issues related to pet ownership in working with clients. Importantly, the specific issues uncovered highlighted how the health and functioning of older adults, in combination with available resources and the home environment, shape the health and wellbeing of people and their pets. The paper highlights the need for programming and community outreach as described by McLennan et al..

The final paper focuses on how the COVID-19 pandemic impacted living with a pet among a sample of adults living in the United States. Rather than examine the direct relationship with the pet, Marcial-Modesto et al. investigated the impact of living with a pet on wellbeing within the context of current relationship status (i.e., partnered or not partnered). The results indicate that during the pandemic, living with a pet was associated with wellbeing but only for people with a partner. The paper raises interesting questions about how demographic factors and historical events shape the impact of pet ownership and our relationships with pets.

The articles in this issue call for greater diverse representation in the field of human-animal interaction. Certainly, this includes demographics such as gender, race, and ethnicity as well as culture, geographic locations, and greater inclusion of non-English speaking populations. To fully understand the human-animal bond, we also need to expand beyond the person-pet dyad. Future studies should involve perspectives from professionals, practitioners, and representatives of organizations including volunteers. These studies also highlight the value of both quantitative and qualitative methodologies. There will always be a need for measuring constructs and outcome measures, but there is also a need to capture the lived experience. The question of directionality (e.g., does pet ownership impact health and wellbeing or do health and wellbeing impact pet ownership) will remain unanswered until longitudinal studies are conducted. Large data sets have begun to include items addressing pet ownership, but none were designed to focus on this topic. Finally, measuring pet ownership with a dichotomous variable is limiting. Not only does it exclude the central role of the human-animal bond, it also overlooks the characteristics of the person and the pet and how they interact in different contexts. Each of these articles illustrate that these characteristics and interactions must be included in studies of pet ownership.

Taken together, this collection of articles provide evidence that our relationships with our pets are influenced by the same multitude of factors that shape our individual lives. The social determinants that impact the health, resources, and wellbeing of people impact the lives of their pets in complex ways that merit attention in further studies. Importantly, the papers indicate that assistance with pet care is needed and appreciated by diverse groups of pet owners not limited to older adults or those with functional limitations or financial barriers. Overall, the results of these four papers provide a nuanced view of the important factors that may shape living with a pet in adulthood.

## Author contributions

JB: Writing – original draft, Writing – review & editing. CC: Writing – review & editing. PY: Writing – review & editing. CO: Writing – review & editing.

